# Supplementary material for: Prevalence of physical violence against people in insecure migration status: A systematic review and meta-analysis
Source: PLoS One. 2024 Mar 27;19(3):e0300189. doi: 10.1371/journal.pone.0300189 (PMC10971783; doi:10.1371/journal.pone.0300189)
Supplement: S1 Appendix — (PDF) [file pone.0300189.s001.pdf]

Citation

Alexandria Innes, Estela Barbosa, Elizabeth Cook, Jessica Corsi, Riikka Kotanen, Sally McManus, Sylvia Walby. Prevalence of violence experienced by people with insecure immigration status, and perceptions of association with immigration control.. PROSPERO 2021 CRD42021268772 Available from: https://www.crd.york.ac.uk/prospero/display_record.php?ID=CRD42021268772

Review question

What is the prevalence of violence experienced by people with insecure immigration status?

Violence causes injury and is related to mental health conditions such as post-traumatic stress and depression. The World Health Organization's 2002 Report of Violence and Health states 'where violence persists, health is compromised.'

Sub questions of the review ask:

1. What is the reported prevalence of different types of violence experienced by people with insecure migration statuses?

2. What types of violence do people in insecure immigration status report experiencing?

3. Do people in insecure migration status who experience violence perceive that to be a consequence of their migration status?

Searches

This review uses a three-stage search strategy.

Stage 1: Electronic database searches

The following databases will be searched: Political Science Complete, SocINDEX, Social Policy and Practice, EMBASE and Web of Science. These databases will be searched for qualitative, quantitative and mixed-methods studies of violence against migrants in state custody, the use of physical enforcement policies (such as pushbacks, restraints in certain settings, forced tranquilizer use), or violence that occurs as a perceived consequence of immigration control policies.

Stage 2: Backwards and forwards citation tracking of included studies using Google Scholar and Web of Science.

Stage 3: Consultations with external networks and advisory boards:

Additional studies will be identified with guidance from the review team and collaborators. Expert recommendations will be sought by authors of key research in the field, and members of the research consortium under the UK Prevention Research Partnership who have relevant expertise. The scoping document and preliminary search results will also be presented at the American Political Science Association Annual Convention for additional feedback and review.

Types of study to be included

We will include:

Quantitative studies that estimate prevalence of any form of violence towards any sub-group of people in insecure migration status and quantitative and qualitative studies that document experiences and perceptions of violence towards groups in insecure migration status. All studies will be peer-reviewed papers;

where violence is either official or de facto state policy, is a verifiable outcome of state policy, or is carried out by an agent of the state in relation to their work for the state, and where violence is directed towards any population in insecure migration status as defined in point 19.

We will exclude:

Conceptual or theoretical studies without empirical findings; or reporting violence pre-1990; studies that measure violence in a subpopulation that includes people with insecure migration status but does not disaggregate by migration status (eg. violence against prison inmates).

Condition or domain being studied

The population being studied is migrants with insecure immigration status and the condition being studied is the experience of violence while in insecure status. Violence causes injury and contributes to health conditions such a post-traumatic stress and depression . Migrants may be in many settings and stages, including on global migration routes and journeys, or after arrival in a receiving state before immigration status is obtained, or in a state in a particular immigration status that is insecure (that may not reflect actual circumstances, or may include a No Recourse to Public Funds rule, or similar), or following a declined petition for immigration.

The aims of the research are:

i) to estimate the prevalence of violence experienced by people with insecure immigration status while an insecure migrant;

ii) to explore whether people with insecure immigration status who experience violence believe that to be a consequence of their immigration status.

Participants/population

Inclusion:

At the time of experiencing violence: people without any current immigration status (having left their country of citizenship or habitual residence); people in the midst of an application for asylum or refugee status or another protected status; people whose status has lapsed or who have overstayed a visa; people who have not continued to fulfil all of the conditions of their status; people whose status has changed but they have not informed the immigration authorities or applied to change it; people who are in a status that does not reflect their current circumstances; people with No Recourse to Public Funds (UK) or the equivalent in other jurisdictions; people who have a particular condition tied to their visa (such as being tied to a particular employer or family relationship).

Exclusion: dual citizens who are residing in a country of their citizenship under conditions that do / did not threaten that citizenship at the time of experiencing violence; lawful permanent residents who are living in conditions that do / did not threaten their status at the time of experiencing violence.

Intervention(s), exposure(s)

The condition of violence will be studied in connection to the exposure to living with insecure migration status.

Comparator(s)/control

Not applicable

Context

Studies of violence experienced by people with insecure immigration status or who do not have immigration

status will be included with no limitation on study setting. The main objective of the study must be to estimate prevalence of (a form of) violence that can be generalised to a defined population in insecure migration status (quantitative) or to document experiences of violence (quantitative and qualitative).

Main outcome(s)

The main outcome of the review is to estimate prevalence of violence experienced by people in insecure immigration status.

Estimates will be produced at a subgroup level because of high levels of heterogeneity in the group of migrants with insecure status. Subgroups will be determined by the available data. The numerator will be the number of migrants in a given category reporting violence and the denominator will be the number of migrants in that category. For example, prevalence of migrants reported by detained migrants: the denominator would be estimate of detained migrants in the reporting period, and the numerator would be the detained migrants who reported violence.

Measures of effect

Not applicable

Additional outcome(s)

A secondary outcome of interest includes a thematic synthesis of qualitative data indicating perceptions of why violence has occurred. The particular association of interest is whether migrants in insecure status who experience violence believe that violence to be related to their immigration status.

Data extraction (selection and coding)

Eligible studies will be identified as detailed in the search strategy. Citations will be transferred to Endnote to remove duplicates. The de-duplicated citations will be uploaded into Rayyan. Titles and abstracts of these studies will be screened according to the inclusion and exclusion criteria independently by two reviewers: the first reviewer will check the whole body. The second reviewer will screen 20% or 250 abstracts (whichever is lower), with the expectation of a minimum of 95% agreement. Any abstract subject to disagreement will be included at this stage.

The full-text of studies included at this stage will be screened against the inclusion and exclusion criteria. A second reviewer will screen 20% or 250 full-texts (whichever is lower with the expectation of a minimum of 95% agreement). Any disagreement will be resolved by discussion, or with reference to a third reviewer if consensus cannot be reached.

Citation tracking and reference list screening will be conducted for retained studies and added at this stage. Also added at this stage will be eligible sources identified by expert recommendations.

Data will be extracted according to a standardised extraction table. This will include: date of publication, date of data collection, host country, defined population, methods, association between violence and immigration status (official or de facto policy; interpersonal violence carried out by an agent of the state in relation to their work for the state; interpersonal violence perceived to be associated with immigration status); specific violence form, perpetrator; subjects, any available disaggregated characteristics of the subjects (sex, ethnicity, nationality, immigration status) . Any measurement of prevalence or other quantitative measures of violence will be recorded. The second reviewer will check the extracted data. If necessary, the authors or owners of the study in question will be contacted for additional information.

Risk of bias (quality) assessment

Using English-language only sources will potentially create bias that defaults interest to English-speaking world.

In cases where there are multiple sources of data from the same country or population, all sources will be included if they meet the inclusion criteria because data will be analysed by sub-groups of insecure migrants,

therefore one study will not automatically override another, and the expectation is that data will bias towards small n studies.

Research will be assessed for internal validity using a standardised quality assessment tool. The selected tool is HTA Initiative #13 Standard Quality Assessment Criteria for Evaluating Primary Research Papers from a Variety of Fields, which offers separate tools for qualitative and quantitative studies while allowing for some consistency evaluating both qualitative and quantitative research.

Strategy for data synthesis

Quantitative Data

Estimates of prevalence will be produced at the level of sub-groups of migrants in insecure status because of high levels of heterogeneity within the data. It is anticipated that quantitative data will be synthesized with a narrative approach due to aforementioned heterogeneity.

Subgroups will be determined by the available data. The numerator will be the number of migrants in a given category reporting violence and the denominator will be the number of migrants in that category. For example, prevalence of migrants reported by detained migrants; the denominator would be the estimate of detained migrants and the numerator would be detained migrants who reported violence.

Meta analysis is not anticipated because of high levels of heterogeneity in the data although if the data permits it will be considered. A minimum of 5 estimates will be required for meta-analysis. The I^2 statistic will be used to assess heterogeneity and the calculation will be conducted in SDATA.

Qualitative Data

Qualitative data will be synthesised thematically. The first reviewer will code data using NVIVO for the type and form of violence, the perpetrators and subject(s) of violence, and any attributed cause of violence, and the attributor (migrant or health / social services professional). This coding will be repeated by the second reviewer. Analysis of will be reported narratively.

Mixed Methods Data

Data will be drawn from all studies to generate a typology of violence table. Data will be coded by the first reviewer for type and form of violence, country, and subject. This data will be organised by country and analysed according to various commonalities and comparators across country (eg. regime type, economic indicators, net migration, immigration numbers). The analysis will be reported narratively.

Analysis of subgroups or subsets

See Strategy for data synthesis

Contact details for further information

Alexandria Innes
alexandria.innes@city.ac.uk

Organisational affiliation of the review

City, University of London
www.city.ac.uk

Review team members and their organisational affiliations

Dr Alexandria Innes. City, University of London
Dr Estela Barbosa. City, University of London
Dr Elizabeth Cook. City, University of London
Dr Jessica Corsi. City, University of London

Dr Riikka Kotanen. City, University of London
Ms Sally McManus. City, University of London
Professor Sylvia Walby. City, University of London

Collaborators

Dr Sian Oram. King's College London

Type and method of review

Narrative synthesis, Synthesis of qualitative studies, Systematic review

Anticipated or actual start date

15 September 2021

Anticipated completion date

31 August 2022

Funding sources/sponsors

City, University of London

UK Prevention Research Partnership (PRP)

Grant number(s)

State the funder, grant or award number and the date of award

UK PRP Consortium Award MR/V049879/1

Conflicts of interest

Language

English

Country

England

Stage of review

Review Ongoing

Subject index terms status

Subject indexing assigned by CRD

Subject index terms

Emigrants and Immigrants; Emigration and Immigration; Humans; Perception; Prevalence; Violence

Date of registration in PROSPERO

31 August 2021

Date of first submission

29 August 2021

Stage of review at time of this submission

Stage	Started	Completed
Preliminary searches	Yes	No
Piloting of the study selection process	No	No
Formal screening of search results against eligibility criteria	No	No
Data extraction	No	No
Risk of bias (quality) assessment	No	No
Data analysis	No	No

The record owner confirms that the information they have supplied for this submission is accurate and complete and they understand that deliberate provision of inaccurate information or omission of data may be construed as scientific misconduct.
The record owner confirms that they will update the status of the review when it is completed and will add publication details in due course.

Versions

31 August 2021
