# Supplementary material for: Prevalence of physical violence against people in insecure migration status: A systematic review and meta-analysis
Source: PLoS One. 2024 Mar 27;19(3):e0300189. doi: 10.1371/journal.pone.0300189 (PMC10971783; doi:10.1371/journal.pone.0300189)
Supplement: S2 Appendix — (PDF) [file pone.0300189.s002.pdf]

Paper Eligibility Criteria

Prevalence of violence experienced by people with insecure immigration status

PEO: Participants = migrants / immigrants; Exposure = insecure immigration status; Outcome = violence.

	**Yes – include**	**No – exclude**
**Report Type**<br>Is it a peer-reviewed journal article?	Peer-reviewed academic journal articles/	Book reviews<br>Reports<br>Conference papers<br>Systematic reviews (but use for additional source articles)<br>Conceptual or theoretical development with no empirical study<br>Magazine articles<br>Dissertations<br>Editorial<br>Non-English language report<br><br>**Reason for Exclusion:**<br>**Wrong Report Type**
**Study Type**<br>Is it a primary study with a quantitative or mixed methods research design?	Quantitative research (cohort, cross-sectional, case-control studies).<br>Mixed-methods reports that reflect the above criteria in quantitative element.	Theoretical or conceptual development with no primary data.<br><br>**Reason for Exclusion:**<br>**Wrong Study Type**
**Participant Population**<br>Are migrants included in the study population.	Study includes migrants or immigrants of any age or gender.	No migrants or immigrants are included in the study.

		**Reason for exclusion:**<br>**Wrong population type**
**Exposure**<br>Insecure immigration status. Are all or some participants people in insecure immigration status at the time of experiencing violence? Is data disaggregated by immigration status in the case of populations that include both secure and insecure statuses?	People without any current immigration status (having left their country of citizenship or habitual residence);<br>people in the midst of an application for asylum or refugee status or another protected status;<br>people whose status has lapsed or who have overstayed a visa;<br>people who have not continued to fulfil all of the conditions of their status;<br>people whose status has changed but they have not informed the immigration authorities or applied to change it;<br>people who are in a status that does not reflect their current circumstances;<br>people with No Recourse to Public Funds (UK) or the equivalent in other jurisdictions;<br>people who have a particular condition tied to their visa (such as being tied to a particular employer or family relationship)	Dual citizens who are residing in a country of their citizenship under conditions that do / did not threaten that citizenship at the time of experiencing violence;<br>lawful permanent residents who are living in conditions that do / did not threaten their status at the time of experiencing violence;<br>people who were not in insecure status when violence occurred (eg. Violence was experienced in home country as motivator of migration);<br>studies where data includes both secure and insecure statuses but data is not disaggregated by insecure migration status (eg 'foreign born' is not evidence of insecure status);<br>people who are in insecure status but are only perpetrators of violence.<br><br>**Reason for exclusion:**<br>**Wrong exposure**
**Outcome**<br>Interpersonal violence	Quantitative measures of direct physical interpersonal violence, including things such as assault, rape, torture as defined in Article 1 of the Convention Against Torture.<br>Policies that involve physical coercion, such as forcible restraint, force feeding, forced use of tranquilizers.	Studies that do not include an outcome of direct physical interpersonal violence.<br>Studies of structural violence such as poverty, food insecurity, homelessness, destitution that do not include specific examples or measurements of interpersonal / physical violence.<br>Historical violence that occurred pre 1990.

		**Reason for exclusion:**<br>**Wrong Outcome**
**Notes:**<br>Eligibility criteria = hierarchy – begin with study type; work downwards until exclusion<br>1st exclusion criteria reached must be recorded as reason for exclusion in Rayyan (wrong study type, wrong data type, wrong population, no outcomes)		
