# Supplementary material for: Prevalence of physical violence against people in insecure migration status: A systematic review and meta-analysis
Source: PLoS One. 2024 Mar 27;19(3):e0300189. doi: 10.1371/journal.pone.0300189 (PMC10971783; doi:10.1371/journal.pone.0300189)
Supplement: S3 Appendix — (PDF) [file pone.0300189.s003.pdf]

Search strategy

Political Science Complete and SocINDEX were searched via Ebscohost. The database coverage was 1944 to the present and the databases were searched on 31 May 2023.

S104	S48 AND S84 AND S103	**Expanders** – Apply equivalent subjects<br>**Search modes** – Boolean / Phrase
S103	S86 OR S88 OR S89 OR S90 OR S92 OR S94 OR S96 OR S98 OR S100 OR S102	**Expanders** - Apply equivalent subjects<br>**Search modes** - Boolean/Phrase
S102	AB survey	**Limiters** - Peer Reviewed; Publication Date: 20000101-20231231; Publication Type: Academic Journal<br>**Search modes** - Boolean/Phrase
S101	AB survey	**Limiters** - Peer Reviewed; Publication Date: 20000101-20231231; Publication Type: Academic Journal<br>**Search modes** - Boolean/Phrase
S100	AB rate	**Limiters** - Peer Reviewed; Publication Date: 20000101-20231231; Publication Type: Academic Journal<br>**Search modes** - Boolean/Phrase
S99	AB rate	**Limiters** - Peer Reviewed; Publication Date: 20000101-20231231; Publication Type: Academic Journal<br>**Search modes** - Boolean/Phrase
S98	AB prevalence	**Limiters** - Peer Reviewed; Publication Date: 20000101-20231231; Publication Type: Academic Journal<br>**Search modes** - Boolean/Phrase

S97	AB prevalence	**Limiters** - Peer Reviewed; Publication Date: 20000101-20231231; Publication Type: Academic Journal<br>**Search modes** - Boolean/Phrase
S96	AB "participant observation"	**Limiters** - Peer Reviewed; Publication Date: 20000101-20231231; Publication Type: Academic Journal<br>**Search modes** - Boolean/Phrase
S95	AB "participant observation"	**Limiters** - Peer Reviewed; Publication Date: 20000101-20231231; Publication Type: Academic Journal<br>**Search modes** - Boolean/Phrase
S94	AB interview*	**Limiters** - Peer Reviewed; Publication Date: 20000101-20231231; Publication Type: Academic Journal<br>**Search modes** - Boolean/Phrase
S93	AB interview*	**Limiters** - Peer Reviewed; Publication Date: 20000101-20231231; Publication Type: Academic Journal<br>**Search modes** - Boolean/Phrase
S92	AB incidence	**Limiters** - Peer Reviewed; Publication Date: 20000101-20231231; Publication Type: Academic Journal<br>**Search modes** - Boolean/Phrase
S91	AB incidence	**Limiters** - Peer Reviewed; Publication Date: 20000101-20231231; Publication Type: Academic Journal<br>**Search modes** - Boolean/Phrase
S90	AB frequency	**Limiters** - Peer Reviewed; Publication Date: 20000101-20231231; Publication Type: Academic Journal<br>**Search modes** - Boolean/Phrase

S89	AB experiential	**Limiters** - Peer Reviewed; Publication Date: 20000101-20231231; Publication Type: Academic Journal<br>**Search modes** - Boolean/Phrase
S88	AB experience	**Limiters** - Peer Reviewed; Publication Date: 20000101-20231231; Publication Type: Academic Journal<br>**Search modes** - Boolean/Phrase
S87	AB experience	**Limiters** - Peer Reviewed; Publication Date: 20000101-20231231; Publication Type: Academic Journal<br>**Search modes** - Boolean/Phrase
S86	AB ethnograph*	**Limiters** - Peer Reviewed; Publication Date: 20000101-20231231; Publication Type: Academic Journal<br>**Search modes** - Boolean/Phrase
S85	AB ethnograph*	**Limiters** - Peer Reviewed; Publication Date: 20000101-20231231; Publication Type: Academic Journal<br>**Search modes** - Boolean/Phrase
S84	S50 OR S51 OR S52 OR S54 OR S56 OR S58 OR S59 OR S60 OR S62 OR S63 OR S64 OR S65 OR S67 OR S69 OR S70 OR S71 OR S72 OR S73 OR S74 OR S75 OR S77 OR S79 OR S81 OR S82 OR S83	**Expanders** - Apply equivalent subjects<br>**Search modes** - Boolean/Phrase
S83	AB "without status"	**Limiters** - Peer Reviewed; Publication Date: 20000101-20231231; Publication Type: Academic Journal<br>**Search modes** - Boolean/Phrase
S82	AB undocumented	**Limiters** - Peer Reviewed; Publication Date: 20000101-20231231; Publication Type: Academic Journal<br>**Search modes** - Boolean/Phrase

S81	AB traffick*	**Limiters** - Peer Reviewed; Publication Date: 20000101-20231231; Publication Type: Academic Journal<br>**Search modes** - Boolean/Phrase
S80	AB traffick*	**Limiters** - Peer Reviewed; Publication Date: 20000101-20231231; Publication Type: Academic Journal<br>**Search modes** - Boolean/Phrase
S79	AB "temporary foreign worker*"	**Limiters** - Peer Reviewed; Publication Date: 20000101-20231231; Publication Type: Academic Journal<br>**Search modes** - Boolean/Phrase
S78	AB "temporary foreign worker*"	**Limiters** - Peer Reviewed; Publication Date: 20000101-20231231; Publication Type: Academic Journal<br>**Search modes** - Boolean/Phrase
S77	AB smuggl*	**Limiters** - Peer Reviewed; Publication Date: 20000101-20231231; Publication Type: Academic Journal<br>**Search modes** - Boolean/Phrase
S76	AB smuggl*	**Limiters** - Peer Reviewed; Publication Date: 20000101-20231231; Publication Type: Academic Journal<br>**Search modes** - Boolean/Phrase
S75	AB refoulement	**Limiters** - Peer Reviewed; Publication Date: 20000101-20231231; Publication Type: Academic Journal<br>**Search modes** - Boolean/Phrase
S74	AB non-native	**Limiters** - Peer Reviewed; Publication Date: 20000101-20231231; Publication Type: Academic Journal<br>**Search modes** - Boolean/Phrase

S73	AB non-immigrant	**Limiters** - Peer Reviewed; Publication Date: 20000101-20231231; Publication Type: Academic Journal<br>**Search modes** - Boolean/Phrase
S72	AB "new comer* OR new-comer*"	**Limiters** - Peer Reviewed; Publication Date: 20000101-20231231; Publication Type: Academic Journal<br>**Search modes** - Boolean/Phrase
S71	AB "naturali$ed citizen*"	**Limiters** - Peer Reviewed; Publication Date: 20000101-20231231; Publication Type: Academic Journal<br>**Search modes** - Boolean/Phrase
S70	AB "migration detention"	**Limiters** - Peer Reviewed; Publication Date: 20000101-20231231; Publication Type: Academic Journal<br>**Search modes** - Boolean/Phrase
S69	AB migrant*	**Limiters** - Peer Reviewed; Publication Date: 20000101-20231231; Publication Type: Academic Journal<br>**Search modes** - Boolean/Phrase
S68	AB migrant*	**Limiters** - Peer Reviewed; Publication Date: 20000101-20231231; Publication Type: Academic Journal<br>**Search modes** - Boolean/Phrase
S67	AB "irregular migra*"	**Limiters** - Peer Reviewed; Publication Date: 20000101-20231231; Publication Type: Academic Journal<br>**Search modes** - Boolean/Phrase
S66	AB "irregular migra*"	**Limiters** - Peer Reviewed; Publication Date: 20000101-20231231; Publication Type: Academic Journal<br>**Search modes** - Boolean/Phrase

S65	AB incomer*	**Limiters** - Peer Reviewed; Publication Date: 20000101-20231231; Publication Type: Academic Journal<br>**Search modes** - Boolean/Phrase
S64	AB "immigration removal"	**Limiters** - Peer Reviewed; Publication Date: 20000101-20231231; Publication Type: Academic Journal<br>**Search modes** - Boolean/Phrase
S63	AB "immigration detention"	**Limiters** - Peer Reviewed; Publication Date: 20000101-20231231; Publication Type: Academic Journal<br>**Search modes** - Boolean/Phrase
S62	AB immigration	**Limiters** - Publication Date: 20000101-20231231; Publication Type: Academic Journal<br>**Search modes** - Boolean/Phrase
S61	AB immigration	**Limiters** - Publication Date: 20000101-20231231; Publication Type: Academic Journal<br>**Search modes** - Boolean/Phrase
S60	AB immigrant*	**Limiters** - Peer Reviewed; Publication Date: 20000101-20231231; Publication Type: Academic Journal<br>**Search modes** - Boolean/Phrase
S59	AB foreign-born	**Limiters** - Peer Reviewed; Publication Date: 20000101-20231231; Publication Type: Academic Journal<br>**Search modes** - Boolean/Phrase
S58	AB foreigner*	**Limiters** - Peer Reviewed; Publication Date: 20000101-20231231; Publication Type: Academic Journal<br>**Search modes** - Boolean/Phrase

S57	AB foreigner*	**Limiters** - Peer Reviewed; Publication Date: 20000101-20231231; Publication Type: Academic Journal<br>**Search modes** - Boolean/Phrase
S56	AB "forced migration"	**Limiters** - Peer Reviewed; Publication Date: 20000101-20231231; Publication Type: Academic Journal<br>**Search modes** - Boolean/Phrase
S55	AB "forced migration"	**Limiters** - Peer Reviewed; Publication Date: 20000101-20231231; Publication Type: Academic Journal<br>**Search modes** - Boolean/Phrase
S54	AB diaspora	**Limiters** - Peer Reviewed; Publication Date: 20000101-20231231; Publication Type: Academic Journal<br>**Search modes** - Boolean/Phrase
S53	AB diaspora	**Limiters** - Peer Reviewed; Publication Date: 20000101-20231231; Publication Type: Academic Journal<br>**Search modes** - Boolean/Phrase
S52	AB deport*	**Limiters** - Peer Reviewed; Publication Date: 20000101-20231231; Publication Type: Academic Journal<br>**Search modes** - Boolean/Phrase
S51	AB "asylum seeker*"	**Limiters** - Peer Reviewed; Publication Date: 20000101-20231231; Publication Type: Academic Journal<br>**Search modes** - Boolean/Phrase
S50	AB alien	**Limiters** - Peer Reviewed; Publication Date: 20000101-20231231; Publication Type: Academic Journal<br>**Search modes** - Boolean/Phrase

S49	AB alien	**Limiters** - Peer Reviewed; Publication Date: 20000101-20231231; Publication Type: Academic Journal<br>**Search modes** - Boolean/Phrase
S48	S2 OR S4 OR S6 OR S8 OR S10 OR S12 OR S15 OR S16 OR S18 OR S20 OR S23 OR S25 OR S27 OR S30 OR S32 OR S34 OR S36 OR S38 OR S40 OR S42 OR S43 OR S47	**Expanders** - Apply equivalent subjects<br>**Search modes** - Boolean/Phrase
S47	AB "violent crime"	**Limiters** - Peer Reviewed; Publication Date: 20000101-20231231; Publication Type: Academic Journal<br>**Search modes** - Boolean/Phrase
S46	AB "violent crime"	**Limiters** - Peer Reviewed; Publication Date: 20000101-20231231; Publication Type: Academic Journal<br>**Search modes** - Boolean/Phrase
S45	AB "violent crime"	**Limiters** - Peer Reviewed; Publication Date: 20000101-20231231; Publication Type: Academic Journal<br>**Search modes** - Boolean/Phrase
S44	AB "violence crime"	**Limiters** - Peer Reviewed; Publication Date: 20000101-20231231; Publication Type: Academic Journal<br>**Search modes** - Boolean/Phrase
S43	AB violence	**Limiters** - Peer Reviewed; Publication Date: 20000101-20231231; Publication Type: Academic Journal<br>**Search modes** - Boolean/Phrase
S42	AB torture	**Limiters** - Peer Reviewed; Publication Date: 20000101-20231231; Publication Type: Academic Journal<br>**Search modes** - Boolean/Phrase

S41	AB torture	**Limiters** - Peer Reviewed; Publication Date: 20000101-20231231; Publication Type: Academic Journal<br>**Search modes** - Boolean/Phrase
S40	AB "sexual violence"	**Limiters** - Peer Reviewed; Publication Date: 20000101-20231231; Publication Type: Academic Journal<br>**Search modes** - Boolean/Phrase
S39	AB "sexual violence"	**Limiters** - Peer Reviewed; Publication Date: 20000101-20231231; Publication Type: Academic Journal<br>**Search modes** - Boolean/Phrase
S38	AB "sexual assault"	**Limiters** - Peer Reviewed; Publication Date: 20000101-20231231; Publication Type: Academic Journal<br>**Search modes** - Boolean/Phrase
S37	AB "sexual assault"	**Limiters** - Peer Reviewed; Publication Date: 20000101-20231231; Publication Type: Academic Journal<br>**Search modes** - Boolean/Phrase
S36	AB rape	**Limiters** - Peer Reviewed; Publication Date: 20000101-20231231; Publication Type: Academic Journal<br>**Search modes** - Boolean/Phrase
S35	AB rape	**Limiters** - Peer Reviewed; Publication Date: 20000101-20231231; Publication Type: Academic Journal<br>**Search modes** - Boolean/Phrase
S34	AB punishment	**Limiters** - Peer Reviewed; Publication Date: 20000101-20231231; Publication Type: Academic Journal<br>**Search modes** - Boolean/Phrase

S33	AB punishment	**Limiters** - Peer Reviewed; Publication Date: 20000101-20231231; Publication Type: Academic Journal<br>**Search modes** - Boolean/Phrase
S32	AB persecution	**Limiters** - Peer Reviewed; Publication Date: 20000101-20231231; Publication Type: Academic Journal<br>**Search modes** - Boolean/Phrase
S31	AB persecution	**Limiters** - Peer Reviewed; Publication Date: 20000101-20231231; Publication Type: Academic Journal<br>**Search modes** - Boolean/Phrase
S30	AB mistreatment	**Limiters** - Peer Reviewed; Publication Date: 20000101-20231231; Publication Type: Academic Journal<br>**Search modes** - Boolean/Phrase
S29	AB mistreatment	**Limiters** - Peer Reviewed; Publication Date: 20000101-20231231; Publication Type: Academic Journal<br>**Search modes** - Boolean/Phrase
S28	AB mistreatment	**Limiters** - Peer Reviewed; Publication Date: 20000101-20231231; Publication Type: Academic Journal<br>**Search modes** - Boolean/Phrase
S27	AB maltreatment	**Limiters** - Peer Reviewed; Publication Date: 20000101-20231231; Publication Type: Academic Journal<br>**Search modes** - Boolean/Phrase
S26	AB maltreatment	**Limiters** - Peer Reviewed; Publication Date: 20000101-20231231; Publication Type: Academic Journal<br>**Search modes** - Boolean/Phrase

S25	AB killing	**Limiters** - Peer Reviewed; Publication Date: 20000101-20231231<br>**Search modes** - Boolean/Phrase
S24	AB killing	**Limiters** - Peer Reviewed; Publication Date: 20000101-20231231<br>**Search modes** - Boolean/Phrase
S23	AB insecurity	**Limiters** - Peer Reviewed; Publication Date: 20000101-20231231; Publication Type: Academic Journal<br>**Search modes** - Boolean/Phrase
S22	AB insecurity	**Limiters** - Peer Reviewed; Publication Date: 20000101-20231231; Publication Type: Academic Journal<br>**Search modes** - Boolean/Phrase
S21	insecurity	**Search modes** - Boolean/Phrase
S20	AB inhumane	**Limiters** - Publication Date: 20000101-20231231; Publication Type: Academic Journal<br>**Search modes** - Boolean/Phrase
S19	AB inhumane	**Limiters** - Publication Date: 20000101-20231231; Publication Type: Academic Journal<br>**Search modes** - Boolean/Phrase
S18	AB harm	**Limiters** - Peer Reviewed; Publication Date: 20000101-20231231; Publication Type: Academic Journal<br>**Search modes** - Boolean/Phrase
S17	AB harm	**Limiters** - Peer Reviewed; Publication Date: 20000101-20231231; Publication Type: Academic Journal<br>**Search modes** - Boolean/Phrase
S16	AB "gang violence"	**Limiters** - Peer Reviewed; Publication Date: 20000101-20231231; Publication Type: Academic Journal

		**Search modes** - Boolean/Phrase
S15	AB force	**Limiters** - Peer Reviewed; Publication Date: 20000101-20231231; Publication Type: Academic Journal<br>**Search modes** - Boolean/Phrase
S14	AB force	**Limiters** - Peer Reviewed; Publication Date: 20000101-20231231; Publication Type: Academic Journal<br>**Search modes** - Boolean/Phrase
S13	AB force	**Limiters** - Peer Reviewed; Publication Date: 20000101-20231231; Publication Type: Academic Journal<br>**Search modes** - Boolean/Phrase
S12	AB exploitation	**Limiters** - Peer Reviewed; Publication Date: 20000101-20231231; Publication Type: Academic Journal<br>**Search modes** - Boolean/Phrase
S11	AB exploitation	**Limiters** - Peer Reviewed; Publication Date: 20000101-20231231; Publication Type: Academic Journal<br>**Search modes** - Boolean/Phrase
S10	AB cruelty	**Limiters** - Peer Reviewed; Publication Date: 20000101-20231231; Publication Type: Academic Journal<br>**Search modes** - Boolean/Phrase
S9	AB cruelty	**Limiters** - Peer Reviewed; Publication Date: 20000101-20231231; Publication Type: Academic Journal<br>**Search modes** - Boolean/Phrase
S8	AB coercion	**Limiters** - Peer Reviewed; Publication Date: 20000101-20231231; Publication Type: Academic Journal

		**Search modes** - Boolean/Phrase
S7	AB coercion	**Limiters** - Peer Reviewed; Publication Date: 20000101-20231231; Publication Type: Academic Journal<br>**Search modes** - Boolean/Phrase
S6	AB attack	**Limiters** - Peer Reviewed; Publication Date: 20000101-20231231; Publication Type: Academic Journal<br>**Search modes** - Boolean/Phrase
S5	AB attack	**Limiters** - Peer Reviewed; Publication Date: 20000101-20231231; Publication Type: Academic Journal<br>**Search modes** - Boolean/Phrase
S4	AB assault	**Limiters** - Peer Reviewed; Publication Date: 20000101-20231231; Publication Type: Academic Journal<br>**Search modes** - Boolean/Phrase
S3	AB assault	**Limiters** - Peer Reviewed; Publication Date: 20000101-20231231; Publication Type: Academic Journal<br>**Search modes** - Boolean/Phrase
S2	AB abuse	**Limiters** - Peer Reviewed; Publication Date: 20000101-20231231; Publication Type: Academic Journal<br>**Search modes** - Boolean/Phrase
S1	AB abuse	**Limiters** - Peer Reviewed; Publication Date: 20000101-20231231; Publication Type: Academic Journal<br>**Search modes** - Boolean/Phrase

Embase and Social Policy and Practice were searched using Ovid. Database coverage was 1974 (Embase) and 1981 (Social Policy and Practice) to the present, and the databases were searched on 31 May 2023.

1.	"immigration removal".ab.
2.	limit 1 to yr="2000 - 2023"
3.	assault.ab.
4.	limit 3 to yr="2000 - 2023"
5.	attack.ab.
6.	limit 5 to yr="2000 - 2023"
7.	coercion.ab.
8.	limit 7 to yr="2000 - 2023"
9.	cruelty.ab.
10.	limit 9 to yr="2000 - 2023"
11.	exploitation.ab.
12.	limit 11 to yr="2000 - 2023"
13.	force.ab.
14.	limit 13 to yr="2000 - 2023"
15.	"gang violence".ab.
16.	limit 15 to yr="2000 - 2023"
17.	harm.ab.

18.	limit 17 to yr="2000 - 2023"
19.	"ill treatment".ab.
20.	limit 19 to yr="2000 - 2023"
21.	insecurity.ab.
22.	limit 21 to yr="2000 - 2023"
23.	killing.ab.
24.	limit 23 to yr="2000 - 2023"
25.	mistreatment.ab.
26.	limit 25 to yr="2000 - 2023"
27.	persecution.ab.
28.	limit 27 to yr="2000 - 2023"
29.	punishment.ab.
30.	limit 29 to yr="2000 - 2023"
31.	rape.ab.
32.	limit 31 to yr="2000 - 2023"
33.	"sexual assault".ab.
34.	limit 33 to yr="2000 - 2023"
35.	"sexual violence".ab.
36.	limit 35 to yr="2000 - 2023"

37.	torture.ab.
38.	limit 37 to yr="2000 - 2023"
39.	violence.ab.
40.	limit 39 to yr="2000 - 2023"
41.	"violent crime".ab.
42.	limit 41 to yr="2000 - 2023"
43.	4 or 6 or 8 or 10 or 12 or 14 or 16 or 18 or 20 or 22 or 24 or 26 or 28 or 30 or 32 or 34 or 36 or 38 or 40 or 42
44.	"asylum seeker".ab.
45.	limit 44 to yr="2000 - 2023"
46.	border*.ab.
47.	limit 46 to yr="2000 - 2023"
48.	citizenship.ab.
49.	limit 48 to yr="2000 - 2023"
50.	deport*.ab.
51.	limit 50 to yr="2000 - 2023"
52.	diaspora.ab.
53.	limit 52 to yr="2000 - 2023"
54.	displacement.ab.
55.	limit 54 to yr="2000 - 2023"

56.	"forced migration".ab.
57.	limit 56 to yr="2000 - 2023"
58.	"human smuggling".ab.
59.	limit 58 to yr="2000 - 2023"
60.	"human trafficking".ab.
61.	limit 60 to yr="2000 - 2023"
62.	immigrant*.ab.
63.	limit 62 to yr="2000 - 2023"
64.	immigration.ab.
65.	limit 64 to yr="2000 - 2023"
66.	"immigration detention".ab.
67.	limit 66 to yr="2000 - 2023"
68.	"immigration removal".ab.
69.	limit 68 to yr="2000 - 2023"
70.	"irregular migra*".ab.
71.	limit 70 to yr="2000 - 2023"
72.	migrant*.ab.
73.	limit 72 to yr="2000 - 2023"
74.	"migration detention".ab.

75.	limit 74 to yr="2000 - 2023"
76.	mobility.ab.
77.	limit 76 to yr="2000 - 2023"
78.	naturalization.ab.
79.	limit 78 to yr="2000 - 2023"
80.	non-immigrant.ab.
81.	limit 80 to yr="2000 - 2023"
82.	refoulement.ab.
83.	limit 82 to yr="2000 - 2023"
84.	refugee*.ab.
85.	limit 84 to yr="2000 - 2023"
86.	smuggl*.ab.
87.	limit 86 to yr="2000 - 2023"
88.	traffick*.ab.
89.	limit 88 to yr="2000 - 2023"
90.	undocumented.ab.
91.	limit 90 to yr="2000 - 2023"
92.	"without status".ab.
93.	limit 92 to yr="2000 - 2023"

94.	45 or 47 or 49 or 51 or 53 or 55 or 57 or 59 or 61 or 63 or 65 or 67 or 69 or 71 or 73 or 75 or 77 or 79 or 81 or 83 or 85 or 87 or 89 or 91 or 93
95.	"case study".ab.
96.	limit 95 to yr="2000 - 2023"
97.	ethnograph*.ab.
98.	limit 97 to yr="2000 - 2023"
99.	experience.ab.
100.	limit 99 to yr="2000 - 2023"
101.	experiential.ab.
102.	limit 101 to yr="2000 - 2023"
103.	frequency.ab.
104.	limit 103 to yr="2000 - 2023"
105.	"focus group*".ab.
106.	limit 105 to yr="2000 - 2023"
107.	incidence.ab.
108.	limit 107 to yr="2000 - 2023"
109.	interview*.ab.
110.	limit 109 to yr="2000 - 2023"
111.	"large n".ab.

112.	limit 111 to yr="2000 - 2023"
113.	"mixed methods".ab.
114.	limit 113 to yr="2000 - 2023"
115.	"participant observation".ab.
116.	limit 115 to yr="2000 - 2023"
117.	prevalence.ab.
118.	limit 117 to yr="2000 - 2023"
119.	qualitative.ab.
120.	limit 119 to yr="2000 - 2023"
121.	quantitative.ab.
122.	limit 121 to yr="2000 - 2023"
123.	rate.ab.
124.	limit 123 to yr="2000 - 2023"
125.	regression.ab.
126.	limit 125 to yr="2000 - 2023"
127.	"semi-structured".ab.
128.	limit 127 to yr="2000 - 2023"
129.	"small n".ab.
130.	limit 129 to yr="2000 - 2023"

131.	statistic*.ab.
132.	limit 131 to yr="2000 - 2023"
133.	survey.ab.
134.	limit 133 to yr="2000 - 2023"
135.	96 or 98 or 100 or 102 or 104 or 106 or 108 or 110 or 112 or 114 or 116 or 118 or 120 or 122 or 124 or 126 or 128 or 130 or 132 or 134
136.	43 and 94 and 135

Web of Science Core Collections, Social Science Research index was searched on 1 June 2023. The database coverage is 1900 to the present.

63	#11 AND #39 AND #62	8:13 AM | Timespan: 2000-01-01to 2023-12-31 (Index Date) | Exact search
62	#40 OR #41 OR #42 OR #43 OR #44 OR #45 OR #46 OR #47 OR #48 OR #49 OR #50 OR #51 OR #52 OR #53 OR #54 OR #55 OR #56 OR #57 OR #58 OR #59 OR #60 OR #61	8:13 AM | Timespan: 2000-01-01to 2023-12-31 (Index Date) | Exact search
61	AB=(punishment)	8:13 AM | Timespan: 2000-01-01to 2023-12-31 (Index Date) | Exact search
60	AB=(rape)	8:13 AM | Timespan: 2000-01-01to 2023-12-31 (Index Date) | Exact search

59	AB=("sexual assault")	8:13 AM | Timespan: 2000-01-01to 2023-12-31 (Index Date) | Exact search
58	AB=(persecution)	8:13 AM | Timespan: 2000-01-01to 2023-12-31 (Index Date) | Exact search
57	AB=(mistreatment)	8:13 AM | Timespan: 2000-01-01to 2023-12-31 (Index Date) | Exact search
56	AB=(maltreatment)	8:13 AM | Timespan: 2000-01-01to 2023-12-31 (Index Date) | Exact search
55	AB=(killing)	8:13 AM | Timespan: 2000-01-01to 2023-12-31 (Index Date) | Exact search
54	AB=(insecurity)	8:13 AM | Timespan: 2000-01-01to 2023-12-31 (Index Date) | Exact search
53	AB=("gang violence")	8:13 AM | Timespan: 2000-01-01to 2023-12-31 (Index Date) | Exact search
52	AB=(harm)	8:13 AM | Timespan: 2000-01-01to 2023-12-31 (Index Date) | Exact search
51	AB=(inhumane)	8:13 AM | Timespan: 2000-01-01to 2023-12-31 (Index Date) | Exact search

50	AB=(force)	8:13 AM | Timespan: 2000-01-01to 2023-12-31 (Index Date) | Exact search
49	AB=(exploitation)	8:13 AM | Timespan: 2000-01-01to 2023-12-31 (Index Date) | Exact search
48	AB=(cruelty)	8:13 AM | Timespan: 2000-01-01to 2023-12-31 (Index Date) | Exact search
47	AB=(coercion)	8:13 AM | Timespan: 2000-01-01to 2023-12-31 (Index Date) | Exact search
46	AB=("sexual violence")	8:13 AM | Timespan: 2000-01-01to 2023-12-31 (Index Date) | Exact search
45	AB=(torture)	8:13 AM | Timespan: 2000-01-01to 2023-12-31 (Index Date) | Exact search
44	AB=(violence)	8:13 AM | Timespan: 2000-01-01to 2023-12-31 (Index Date) | Exact search
43	AB=("violent crime")	8:13 AM | Timespan: 2000-01-01to 2023-12-31 (Index Date) | Exact search

42	AB=(abuse)	8:13 AM | Timespan: 2000-01-01to 2023-12-31 (Index Date) | Exact search
41	AB=(assault)	8:13 AM | Timespan: 2000-01-01to 2023-12-31 (Index Date) | Exact search
40	AB=(attack)	8:13 AM | Timespan: 2000-01-01to 2023-12-31 (Index Date) | Exact search
39	#12 OR #13 OR #14 OR #15 OR #16 OR #17 OR #18 OR #19 OR #20 OR #21 OR #22 OR #23 OR #24 OR #25 OR #26 OR #27 OR #28 OR #29 OR #30 OR #31 OR #32 OR #33 OR #34 OR #35 OR #36 OR #37 OR #38	8:13 AM | Timespan: 2000-01-01to 2023-12-31 (Index Date) | Exact search
38	AB=(traffick*)	8:13 AM | Timespan: 2000-01-01to 2023-12-31 (Index Date) | Exact search
37	AB=(undocumented)	8:13 AM | Timespan: 2000-01-01to 2023-12-31 (Index Date) | Exact search
36	AB=("without status")	8:13 AM | Timespan: 2000-01-01to 2023-12-31 (Index Date) | Exact search
35	AB=("temporary foreign worker*")	8:13 AM | Timespan: 2000-01-01to 2023-12-31 (Index Date) | Exact search
34	AB=(smuggl*)	8:13 AM | Timespan: 2000-01-01to 2023-12-31 (Index Date) | Exact search

33	AB=(refoulement)	8:13 AM | Timespan: 2000-01-01to 2023-12-31 (Index Date) | Exact search
32	AB=(non-native)	8:13 AM | Timespan: 2000-01-01to 2023-12-31 (Index Date) | Exact search
31	AB=(non-immigrant)	8:13 AM | Timespan: 2000-01-01to 2023-12-31 (Index Date) | Exact search
30	AB=("newcomer* OR new-comer*")	8:13 AM | Timespan: 2000-01-01to 2023-12-31 (Index Date) | Exact search
29	AB=("naturali$ed citizen*")	8:13 AM | Timespan: 2000-01-01to 2023-12-31 (Index Date) | Exact search
28	AB=("migration detention")	8:13 AM | Timespan: 2000-01-01to 2023-12-31 (Index Date) | Exact search
27	AB=(migrant*)	8:13 AM | Timespan: 2000-01-01to 2023-12-31 (Index Date) | Exact search
26	AB=("irregular migra*")	8:13 AM | Timespan: 2000-01-01to 2023-12-31 (Index Date) | Exact search

25	AB=(incomer*)	8:13 AM | Timespan: 2000-01-01to 2023-12-31 (Index Date) | Exact search
24	AB=("immigration detention")	8:13 AM | Timespan: 2000-01-01to 2023-12-31 (Index Date) | Exact search
23	AB=("immigration removal")	8:13 AM | Timespan: 2000-01-01to 2023-12-31 (Index Date) | Exact search
22	AB=(immigration)	8:13 AM | Timespan: 2000-01-01to 2023-12-31 (Index Date) | Exact search
21	AB=(immigrant*)	8:13 AM | Timespan: 2000-01-01to 2023-12-31 (Index Date) | Exact search
20	AB=("human trafficking")	8:13 AM | Timespan: 2000-01-01to 2023-12-31 (Index Date) | Exact search
19	AB=("human smuggling")	8:13 AM | Timespan: 2000-01-01to 2023-12-31 (Index Date) | Exact search
18	AB=(foreign-born)	8:13 AM | Timespan: 2000-01-01to 2023-12-31 (Index Date) | Exact search
17	AB=(foreigner*)	8:13 AM | Timespan: 2000-01-01to 2023-12-31 (Index Date) | Exact search

16	Search<br>AB=("forced migration")	8:13 AM | Timespan: 2000-01-01to 2023-12-31 (Index Date) | Exact search
15	AB=(diaspora)	8:13 AM | Timespan: 2000-01-01to 2023-12-31 (Index Date) | Exact search
14	AB=(deport*)	8:13 AM | Timespan: 2000-01-01to 2023-12-31 (Index Date) | Exact search
13	AB=("asylum seeker*")	8:13 AM | Timespan: 2000-01-01to 2023-12-31 (Index Date) | Exact search
12	AB=(alien)	8:13 AM | Timespan: 2000-01-01to 2023-12-31 (Index Date) | Exact search
11	#1 OR #2 OR #3 OR #4 OR #5 OR #6 OR #7 OR #8 OR #9 OR #10	8:13 AM | Timespan: 2000-01-01to 2023-12-31 (Index Date) | Exact search
10	AB=(survey)	8:13 AM | Timespan: 2000-01-01to 2023-12-31 (Index Date) | Exact search
9	AB=(rate)	8:13 AM | Timespan: 2000-01-01to 2023-12-31 (Index Date) | Exact search

8	AB=(interview*)	8:13 AM | Timespan: 2000-01-01to 2023-12-31 (Index Date) | Exact search
7	AB=(prevalence)	8:13 AM | Timespan: 2000-01-01to 2023-12-31 (Index Date) | Exact search
6	AB=("participant observation")	8:13 AM | Timespan: 2000-01-01to 2023-12-31 (Index Date) | Exact search
5	AB=(frequency)	8:13 AM | Timespan: 2000-01-01to 2023-12-31 (Index Date) | Exact search
4	AB=(incidence)	8:13 AM | Timespan: 2000-01-01to 2023-12-31 (Index Date) | Exact search
3	AB=(experiential)	8:13 AM | Timespan: 2000-01-01to 2023-12-31 (Index Date) | Exact search
2	AB=(experience)	8:13 AM | Timespan: 2000-01-01to 2023-12-31 (Index Date) | Exact search
1	AB=(ethnograph*)	8:13 AM | Timespan: 2000-01-01to 2023-12-31 (Index Date) | Exact search
