# Supplementary material for: Prevalence of physical violence against people in insecure migration status: A systematic review and meta-analysis
Source: PLoS One. 2024 Mar 27;19(3):e0300189. doi: 10.1371/journal.pone.0300189 (PMC10971783; doi:10.1371/journal.pone.0300189)
Supplement: S4 Appendix — (PDF) [file pone.0300189.s004.pdf]

Appendix 2: Insecure Immigration Status

(adapted from Innes, A. 2023. "Migration, Vulnerability, and Experiences of Insecurity:

Immigration Status	Examples of Insecurity and Related Precarity
No status/undocumented	Subject to border enforcement (pushbacks, restraint, and detention); subject to removal so tend to avoid contact with social services, police, and healthcare where possible. Gives rise to vulnerability to exploitation in employment and living circumstances.
Asylum seeker	Subject to deterrence policies of the state. Subject to violence in detention facilities, restraint, and removal. Subject to violent border policies, and pushbacks.
Failed asylum seeker	As above, also subject to destitution and removal to unsafe location. Lack recourse to health, social, and specialist services.
Overstayer/lapsed status	Subject to removal, destitution, vulnerable to exploitation, lack recourse to health, social, and specialist services.
Not fulfilling conditions	Subject to removal, destitution, and vulnerable to exploitation. Some scope to regain full visa by fulfilling conditions.
Change in circumstances	Subject to removal, destitution, and vulnerable to exploitation. Some scope to regain full visa by filing change in circumstances.
No recourse to public funds	Enhances vulnerability to exploitation by the person to whom the visa is tied (such as spousal). Lack recourse to health, social, and specialist services. Leaving unsafe circumstances produces vulnerability to destitution.
Status tied to employer	Subject to enforced servitude, unsafe working conditions, and unsafe living conditions with no recourse to report.
Status tied to family	Vulnerable to domestic violence, enforced servitude, financial abuse, and bureaucratic abuse.

Conceptualising Insecure Migration Status" *Social Sciences* 12, no. 10: 540. https://doi.org/10.3390/socsci12100540)
