# Supplementary material for: Prevalence of physical violence against people in insecure migration status: A systematic review and meta-analysis
Source: PLoS One. 2024 Mar 27;19(3):e0300189. doi: 10.1371/journal.pone.0300189 (PMC10971783; doi:10.1371/journal.pone.0300189)
Supplement: S5 Appendix — (PDF) [file pone.0300189.s005.pdf]

Risk Of Bias in Included Studies

Adapted from the Joanna Briggs Assessment Tool for Cross Sectional Studies

	Inclusion criteria defined	Study subjects and setting described	Exposure measured in valid and reliable way	Insecure immigration status measured using objective, standard criteria	Identified confounding factors	Strategies to deal with confounding	Outcomes measured in valid and reliable way	Reports raw data	OR /RR reported with 95% CIs
Arsenijevic Et Al 2017	xx	**	-	**	*	xx	xx	**	N/A
Ben Farhat Et Al 2018	xx	**	-	**	*	xx	-	**	N/A
Bianchi Et Al 2021	xx	**	-	*	xx	N/A	-	*	N/A
Bouhenia Et Al 2017	**	**	*	**	xx	N/A	-	xx	N/A
Bronsino Et Al 2020	**	**	**	**	*	**	-	**	N/A
Coulter Et Al 2020	**	**	*	**	*	xx	-	*	N/A
Dias Et Al 2013	**	**	-	*	xx	N/A	xx	**	N/A
Gezie Et Al 2019	*	**	*	**	*	xx	-	**	**
Gorn Et Al 2023	**	*	-	-	xx	N/A	-	**	N/A
Hadush Et Al 2023	**	**	-	*	xx	N/A	*	*	N/A
Infante Et Al 2012	*	**	**	**	xx	N/A	*	*	N/A
Islam Et Al 2021	**	**	*	*	xx	N/A	*	**	N/A
Jankovic-Rankovic Et Al 2020	xx	**	-	**	*	xx	-	*	N/A

Leyva-Flores Et Al 2019	-	*	-	xx	xx	N/A	-	*	N/A
Logie Et Al 2022	**	**	*	*	xx	N/A	**	**	N/A
Meyer Et Al 2019	**	**	*	-	*	xx	*	**	**
Morof Et Al 2013	**	**	*	**	xx	N/A	*	**	N/A
Nakash Et Al 2015	*	**	-	**	xx	N/A	-	**	N/A
Ogbonnaya Et Al 2015	**	**	-	**	xx	N/A	-	*	**
Okenwa-Emegwa Et Al 2021	*	**	xx	**	xx	N/A	**	*	**
Phillips Et Al 2006	*	*	-	*	xx	N/A	-	xx	xx
Phillips Et Al 2002	*	*	*	**	xx	xx	*	*	N/A
Pocock Et Al 2018	*	**	-	**	xx	N/A	-	**	N/A
Reques Et Al 2020	**	**	*	*	x	xx	*	*	N/A
Scott 2022	xx	**	**	**	xx	N/A	*	*	N/A
Segneri Et Al 2022	xx	**	-	-	xx	N/A	*	**	N/A
Stewart Et Al 2012	**	**	-	**	*	*	-	*	**
Suyanto Et Al 2020	**	xx	-	*	xx	N/A	-	xx	N/A
Vila and Pomeroy 2020	**	**	*	*	x	xx	-	xx	N/A
Vives-Cases Et Al 2014	**	**	*	-	xx	N/A	*	**	**
Zadnik Et Al 2016	**	**	-	*	xx	N/A	*	*	xx

** criterion completely satisfied; * criterion partially satisfied; - unclear or insufficient information; ^{X} criterion partially not satisfied; ^{XX} Criterion not satisfied; N/A Not applicable
