# Supplementary material for: Prevalence of physical violence against people in insecure migration status: A systematic review and meta-analysis
Source: PLoS One. 2024 Mar 27;19(3):e0300189. doi: 10.1371/journal.pone.0300189 (PMC10971783; doi:10.1371/journal.pone.0300189)
Supplement: Attachment — Submitted filename: Manuscript Review Report.docx [file pone.0300189.s006.docx]

**Manuscript Review Report**

**Journal: PLOSONE**

**Manuscript title**: “Prevalence of physical violence against people in insecure migration status: A systematic review and meta-analysis”

**Manuscript number**: PONE-D-23-31719

**General note:** The prevalence of studies was reported inconsistently, necessitating the authors to re-analyze their data (See specific remark beneath the results, however the errors are not limited to the remarks).

**Abstract**

- (line 33) Why was your database searching limited to publications from January 2000 until 31 May 2023? Specify the date for each databases (See PRISMA 2020 for abstracts checklist, item # 4)
- (line 38-39) How did you come to the conclusion that the prevalence of physical violence was higher in men than in women when the interval estimates for the two subgroups (men versus women) overlapping (95% CI: 18.45-52.15 versus 21.42-34.15)? The same question applies to other conclusions and the accompanying interval estimates.
- (line 51) What evidence did you use to conclude that the prevalence of physical violence in the study group is **high**? To make the question clearer, when do we say the physical violence prevalence is **low**?
- Some sentences are vague (difficult to understand).
- Follow the PRISMA 2020 for abstract checklist to write the abstract

**Introduction**

- (line 69) What does the acronym UNODC stand for?
- (line 79) “a similar stipulation elsewhere” (please cite your source)

**Methods**

- (line 126) “The exposure was insecure status.” What is the point of mentioning this given that your study does not measure effect (association)?
- ” Specify the date when each source was last searched or consulted” (see PRISMA 2020 item # 6)
- “Present the full search strategies for all databases, registers and websites, including any filters and limits used”- PRISMA 2020 item checklist, item # 7)

**Results**

- (line 267) Your database search dates span nearly two years (on September 20, 2021 and May 31, 2023). A database search on May 31, 2023 cannot generate the same amount of records as a database search two years earlier. Hundreds of articles on the same topic can be published even in a month. What is your scientific justification for this?
- Provide the outputs of database searches so that other researchers can verify that the results of your searches are repeatable for each of the databases you searched. You can include the search outputs as an annex for each database or report them in the main document.
- For your example: PubMed Search Output: (((under nutrition) OR (malnutrition)) AND ((child) OR (pediatric))) AND ((critically ill) OR (intensive care unit)). If anyone inserts and searches this in PubMed, the result would be 967 on July 11, 2023, at 13:09:48, but it turns out 993 records when searched after about three months on October 23, 2023.
- (line 273) The PRISMA flow diagram presented does not adhere to the updated 2020 guideline.
- The sub-group analyses were unable to reduce heterogeneity. Look for another source of heterogeneity. Conduct meta-regression analysis. Finally, none of the sub-group analyses helped to identify the source of heterogeneity.
- **Sensitivity analysis/influential study** analysis was not done (i.e. PRISMA 2020 item# **20d** not addressed)
- **Publication bias** not addressed (PRISMA 2020 item# 21)
- Why is Reques et al.'s prevalence report in figure-2 91.67% and in figure-3 82.35%? Figures 2 and 3 show the same inconsistency of prevalence for Dias et al.'s 2013, Pockock et al.'s 2018, and many more publications.
- Unnecessary redundancy of phrases observed

**Discussion**

- The first few paragraphs of the discussion are a repetition of the results report
- The pooled prevalence estimate in the result (line 317) is 31.16%, whereas in the discussion (line 390), it is 30.86%
- (line 389) “…35 cross-sectional studies….” is not consistent with “….included 31 studies….” (line 270). Similarly, the number of participants of the included studies?
- Discuss the highest prevalence reported by Reques et al., 2020 and the lowest reported by Vives-Cases et al., 2014.

**Annex**

Figure 1. Consult “PRISMA 2020 flow diagram template for systematic reviews”

Figure 2. What is the "ES" in the upper right hand corner of the figure?

Figure 3: Why is Reques, et al.'s prevalence report 91.67% in figure 2 and 82.35% in figure 3? The same for Dias et al., 2013; Pockock 2018…………etc
