# Supplementary material for: Prevalence of physical violence against people in insecure migration status: A systematic review and meta-analysis
Source: PLoS One. 2024 Mar 27;19(3):e0300189. doi: 10.1371/journal.pone.0300189 (PMC10971783; doi:10.1371/journal.pone.0300189)
Supplement: Attachment — Submitted filename: PLOS-R&R-response.docx [file pone.0300189.s007.docx]

Dear Editors and Anonymous Reviewers,

Thank you for considering our manuscript ‘Prevalence of Physical Violence Against People in Insecure Migration Status: A Systematic Review’ for publication in PLOS One. We are grateful for the reviewer comments, and believe that they have supported the development of this manuscript.

Please find below a detailed table indicating the reviewer responses and the ways we have addressed them in the manuscript. Please note that page numbers in **bold** refer to the manuscript that has changes tracked.

| Reviewer Comments | Authors’ Response |
| --- | --- |
| **Reviewer 1** |  |
| 1.1 Line 62-71: consider citing references for these comments. | We have specified the citations **(lines 75-78)** |
| 1.2 Is there a category that accounts for pre-migratory violence? This could be a risk factor for mental health outcomes. Consider saying something about this even though it is not the population of interest in this study. | The asylum seeker / refugee category definitively includes pre-migratory violence. See **lines 451-454**, **Figure 4**. |
| 1.3 Line 185: The exclusion of other forms of violence is well explained in the context of scientific rigor. The methods and analytical tools appear to be quite thorough. | Thank you for the positive feedback |
| 1.4 Line 334: refers to eleven studies, then the same twelve studies. Is this an error? | Yes, this was an error. Thank you. Eleven is correct and the relevant correction has been made int the text **(line 435)** |
| 1.5 Line 407: I suggest stating the prevalence rate for IPV in insecure migration here so reader can easily compare to the gen pop 23.1%. | Addition is now included (**line 550-551**) |
| 1.6 Line 538: on what basis do you assert that "particularly liberal states" are notoriously secretive? One might argue that no state is open about the violence they perpetrate. Perhaps there is a debate to be had but it strays from the rest of the discussion, so I suggest removing "and particularly liberal states". | We agree this is a bigger theoretical discussion that is unnecessary to invite at this point. Consequently we have removed ‘ and particularly liberal states’ on **line 713**. |
| 1.7 The intro and discussion are true to the study but lacks some engagement of the reader on the clinical implications. Why is it important to study violence? What effect does physical violence have on migrants? No where in the paper is there mention of trauma, mental health or physical health outcomes. You may wish to contract some of the research and policy implications to be more concise and add some empathic, clinical implications in order to contextualize the research and engage readership. | We have added a lead-in paragraph with relevant citations to the ‘Rationale’ section (**lines 96-113)**. |
| **Reviewer 2** |  |
| 2.1 Abstract, methods, please add name of databases. Also, add your checklist for quality appraisal and the methods used for meta-analysis. Add the software and replace the PROSPERO information to the end of the abstract. | All of these have been added into the abstract, **lines 30-64** |
| 2.2 Abstract, results, please add the p-values for all outcomes. | p-values have been added to the abstract, **lines 40-55**. |
| 2.3 Line 87, please remove this heading. | We have decided to keep this heading indicating the rationale for the study. This is in response to comments from Reviewers 1 and 4, asking to elaborate on this section **(line 94)**. |
| 2.4 Line 116-118 are redundant. Please remove from here. | These lines have been removed. |
| 2.5 Line 122, eligibility criteria, this section is too long. Please briefly mention only the inclusion and exclusion criteria. Based on the PRISMA (Items 10a and 10b), the rest of the sentences that contain definitions should be mentioned in the data extraction section. | We have revised this section as suggested. See **lines 147-152 and Appendix 2: Eligibility Criteria**. |
| 2.6 Line 186, type of study, there is no such a subheading in the PRISMA checklist. This information should be mentioned in the eligibility criteria section. | We removed the subheading ‘Type of study’ and moved this information to the eligibility criteria section. |
| 2.7 Lines 189-90 is related to search. Please remove these information from this section. | We have removed this information. |
| 2.8 What is the reason for not searching major databases such as PubMed and Scopus? I think you missed some studies. | The databases were chosen based on advice from information specialists in Health Sciences and Sociology, and author expertise in political science and social sciences. Embase expands on Medline which includes 92% of PubMed. Web of Science was selected to complement the range of databases spanning social and health sciences. We are confident that the search was comprehensive. We have acknowledged that there is a chance some studies were missed due to database selection to the ‘limitations’ section (**lines 646-648**) |
| 2.9 Line 195, the search period and the exact time of the search must be mentioned.  , . | This has been done (**lines 243-245, also see Appendix 3**) |
| 2.10 In my opinion, it is better to merge information sources and search strategy under one heading | We followed PRISMA 2020 checklist for Methods section: item 6. Information sources, item 7. Search strategy. |
| 2.11 In my opinion, Tables 1 and 2 can be added to the article as supplementary files. | Agreed, we moved tables 1 and 2 to supplementary files, **Appendices 3 and 4** . |
| 2.12 Based on PRISMA, please add search strategy for all databases as a supplementary file. This file also include the number of records and time of search. | This has been added.  We have followed PRISMA 2020 explanation and elaboration and uploaded a supplementary table with the search strategy for each database (**Appendix 3**). |
| 2.13 Line 248, please add the version. | The Stata version number has been added, **line 342.** |
| 2.14 What about publication bias? Please add funnel plot for at least one outcome. | For meta-analyses of proportions/prevalences, research suggests that funnel plots are not recommended as they tend to give erroneous results when pooling prevalence data, therefore we have not produced funnel plots for our analyses. E.g.:  Cheema, H. A., Shahid, A., Ehsan, M., & Ayyan, M. (2022). The misuse of funnel plots in meta-analyses of proportions: are they really useful?. *Clinical Kidney Journal*, *15*(6), 1209-1210.  Hunter, J. P., Saratzis, A., Sutton, A. J., Boucher, R. H., Sayers, R. D., & Bown, M. J. (2014). In meta-analyses of proportion studies, funnel plots were found to be an inaccurate method of assessing publication bias. *Journal of clinical epidemiology*, *67*(8), 897-903.  However we have added an acknowledgement of this possibility to the ‘limitations’ section (**lines 655-657**). |
| 2.15 Lines 267-272 needs subheading: "study selection", items 16a and 16b of PRISMA checklist. | We have added a subheading of ‘study selection’, **line 361**. |
| 2.16 Since different tools/questionnaires are used in different studies and the variety of tools can affect the results, I think it is better to add a column to Table 3 and mention the type of tool. Secondly, it is better to add an analysis according to the type of tool to the results. | We have added column Measurement tool to table 3, now titled table 1 **(line 401)**.  Six out of thirty-one studies used a tool, and each study used a different tool. Therefore, we think the potential for robust meta-analysis is limited because of the diversity among tools adopted to measure violence. |
| 2.17 Line 307, please add some percent in this section for your results. | We have added the relevant percentages. |
| 2.18 Please remove subheadings from the discussion section. | It is common for reviews published in PLOS One to specify subsections in the discussion section, at least including Limitations and Conclusion. We have adjusted our subsections to replicate this PLOS One style (**line 492-723**) |
| 2.19 Please add a conclusion. | Following the style of systematic reviews published in PLOS we have added a short conclusion (**Lines 724-736**) |
| 2.20 Line 51, it is not clear enough. How you interpreted that " the prevalence of violence against people in insecure migration status is high". I search the whole manuscript and there is not a category for your statement or not a reference for it. Please clear it. I agree with you that this is a high value, but it is better to have a reference for this statement. | I have removed the words ‘is high and’ from **line 57-58** so it now reads ‘The review finds that the prevalence of violence against people in insecure migration status should be considered a concern for people in a range of insecure statuses.’ |
| **Reviewer 3** |  |
| 3.1 the conclusion of higher/lower prevalence when comparing different sub-groups of study participants was not statistically supported (the compared sub-groups have overlapping confidence intervals). | Reviewer 2 highlighted a similar issue (see comment 2.20). We have revised our prevalence statements throughout the manuscript (**eg on lines 42-55, 430-432, 449-454, 460-465, 489-90**) |
| 3.2 More importantly, the analysis has computational flaws that result in conflicting results (e.g., various prevalence reports for the same study). | Thank you for your comment. The reason for the difference in prevalences in Figure 3 compared to the other figures is because Figure 3 looks at the female and male prevalences separately, whereas the other analyses are for the overall study population (men and women combined). Therefore, for studies that report include a mix of men and women, the prevalence estimate will vary depending on whether the overall prevalence for men and women combined is used, or whether two prevalences (one for men and one for women) are used. For example, in Figure 4, the prevalence for Reques 2020 is 91.67% and is based on all study participants (men and women). In Figure 3, the prevalence is different because it is reported separately for men (94.55%) and women (82.35%) |
| **Reviewer 4** |  |
| 4.1 First, the authors should consider providing specific examples and relevant references in lines 64-71. Although those points are reasonable, their credibility should be strengthened further with evidence from earlier research. | I have specified the references that are in there to each of the points and have added some additional references (**see lines 75-79**). |
| 4.2 the authors may wish to add reference(s) for four broader categories they used in lines 88-89 and afterward. Also, they must mention if those categories are relevant to physical violence only or all forms of violence. If later, they should explain how those categories are useful to distinguish physical violence from others and how those constructs are used in this meta-analytic review. | I have specified the references that are in there to each of the points and have added some additional references (**see lines 97-139**). |
| 4.3 in the background, the authors should strengthen the rationale for this review. A few ideas, such as the current trends in insecure migration globally, their predictors and consequences, and relevance to estimating physical violence, should be discussed, establishing sufficient reasons why this study is needed, or in other words, how this study can help policymakers and practitioners. Those areas should be explored and comprehensively discussed before presenting the aim/purpose of this study. | I have adjusted this in response to reviewer 1, although the level to which they can be ‘comprehensively discussed’ is affected by space limitations. I think the references suffice now to demonstrate the urgency of the problem. **See lines 96-12**0**.** |
| 4.4 the authors should explain why physical violence is studied in this article rather than psychological, social, or other forms of violence. Any case or type of violence is unacceptable. However, we need to justify why we consider or prioritize one type as a focus of this work. | We have added an explanatory sentence (lines **108-112**) |
| 4.5 I admire the efforts of the authors to clarify the lack of definition for insecure immigration status and their proposed constructs that may capture this idea in different population groups who don’t have secure immigration status. However, those categories may not be consistent across contexts. For example, employment-based immigrants vs. undocumented immigrants have notable differences in terms of psychosocial, legal, administrative, and other forms of stressors that may affect their vulnerability to violence. | The sub-group analysis on status type was designed to investigate this. These groups were data-driven. |
| 4.6 specific categories, such as asylum seekers, have different sets of rights and protections in different jurisdictions that may affect their inclusion in insecure status, as mentioned in this study. Their insecurity is generally higher in places of origin rather than where they stayed after voluntary displacement/migration. Those concepts should be clarified either in Table 1 in a separate column and/or within the methods section. Also, having a broader definition of “secure immigration status” with sufficient sources could be helpful. | The definition of asylum seeker and refugee in the literature varied: some studies adopted the legal definition, while others adopted a self-definition of asylum seekers / refugees. We were interested in status type so only included those currently in refugee (usually camp-based) or asylum seeking status so as to rule out people who had entered as a refugee and then gained Lawful Permanent Residence or citizenship. The relative insecurity in country of origin is not of interest to this study as violence pre-migration is excluded, see **Appendix 2**. |
| 4.7 while categorizing the perpetrators, the authors may consider other possible subgroups, such as employers, specific non-state entities, and interpersonal violence that don’t fall in IPV or state-induced violence. | Thank you for this suggestion. Our categorization of all sub-groups, including perpetrators of violence was data-driven, and more categories were not included because the data was not available. |
| 4.8 please provide a detailed explanation of the risk of bias assessment and how studies with varying risks of bias offered similar/different estimates for physical violence. | We revised the risk of bias reporting to align with the PRISMA 2020 statement (items 11 and 18). We added information about how we assessed the risk of bias (**lines 317-340**) and what we found (**lines 404-411). Appendix 5** presents risk for each study by nine domains. |
| 4.9 a funnel plot is necessary to establish whether publication bias was present in this meta-analysis. Please consider using this approach, as publication bias can profoundly impact the credibility and scope of the evidence in meta-research. | Thank you. See response to comment 2.13 above. |
| 4.10 the role of covariates should be examined using a meta-regression approach. Please explain why that was not conducted in this study, and consider adding meta-regression for key variables/covariates of interest, which may enrich this study. | All important covariates/key variables that were available in the data were explored via subgroup analyses, so we don't think there is additional value in meta-regression. |
| 4.11 the discussion sufficiently reflects on the prevalence of physical violence but provides limited insights into why the estimates vary across specific subgroups.  Also, the social determinants of violence and their roles in burden estimates should be briefly discussed. Possible causes of varying estimates across studies and groups should be explained using previous research so that the readers can contextualize the findings of this meta-analytic study. | We have added some clarifying sentences regarding the findings we have on **lines 548-553, 585-586, 604-606**. We have also reiterated the prevalence estimates in the relevant place for ease for the reader.  The social determinants of violence that we discussed include gender and immigration status because these two risk factors were consistently reported across the studies. Other social determinants of violence (e.g., age, socio-economic conditions, social norms, laws/policy/institutions) might have contributed to the varying estimates, but data on these determinants was not available across studies. There was no consistency across studies in terms of variables reported, so we were unable to extract and include in our study.  We have added to the discussion on gender as a social determinant of violence, and reorganised the discussion section to better reflect the organisation of the study **(lines 512-586)**. |
| 4.12 A brief conclusion with key take-away messages of this study would be helpful. | Included, see **lines 724-736** |

Thank you again for your time reviewing this work. Please do contact me at [alexandria.innes@city.ac.uk](mailto:alexandria.innes@city.ac.uk) if there is anything further we can provide.

We will look forward to hearing from you in due course.

Sincerely yours,

Alexandria Innes
