# Supplementary material for: Prevalence of physical violence against people in insecure migration status: A systematic review and meta-analysis
Source: PLoS One. 2024 Mar 27;19(3):e0300189. doi: 10.1371/journal.pone.0300189 (PMC10971783; doi:10.1371/journal.pone.0300189)
Supplement: Attachment — Submitted filename: PLOS-R&R-response-Jan24.docx [file pone.0300189.s008.docx]

School of Policy and Global Affairs

City, University of London, Northampton Square, London EC1V 0HB

30 January 2024

Dear Editors and Anonymous Reviewers,

Thank you again for considering our manuscript ‘Prevalence of Physical Violence Against People in Insecure Migration Status: A Systematic Review’ for publication in PLOS One. We are very grateful for the reviewer comments, and believe that they have supported the development of this manuscript. We were very pleased to have a positive response from Reviewer 2 who considered the manuscript acceptable for publication.

**Reviewer 3** advanced the following query:

We employ two statistical methods to conclude a comparison of two or more statistics, as one is different from (greater or lower than) the other. One is a confidence interval. When the confidence interval excludes the null value or when the two confidence intervals do not overlap, we can conclude that the observed values differ (one is higher or lower than the other). A p-value can also be used to test for statistical significance. A p-value shows the probability of obtaining the observed difference if the null hypothesis of no difference is true.
For example, we may use the p-value of the selected test statistic to assess whether the difference in prevalence for men and women of 7.52% (i.e., 35.30%-27.78%) is attributable to chance alone (random error) or to an actual difference in prevalence among the underlying male and female populations. Similarly, we can create a null hypothesis for the difference (prevalence in males versus prevalence in women) and use a confidence interval to determine whether the observed difference reflects the underlying population difference or not. We cannot conclude that there is a difference between the two populations if the confidence interval contains the stated null value.
Alternatively, for the comparison groups to be different, their confidence intervals must not overlap. In this study, for example, the 95% confidence intervals of 18.45-52.15 for men and 21.42-34.15 for women overlap, rendering the conclusion that men had a larger prevalence than women incorrect. How come men's lower limit of 18.45% prevalence is greater than women's lower limit of 21.42% prevalence?

Fundamentally, both the confidence interval approach and the p-value approach yield the same conclusion for the same data, but this is not maintained in this manuscript under consideration. Except for the absence of clarity of the aforementioned scientific integrity concern, the manuscript is really well written, and I feel it will be very useful in establishing an international intervention approach.

Thank you for the detailed comment. Reviewer 2 asked to add the p-values for all outcomes throughout abstract and results which we did. In response to your comment, we have reconsidered our reporting and adjusted the language to ensure that we are very clear that there is no statistically significant difference between the prevalence estimates.

You will find the changes to reflect this tracked at lines: **41-52, 340-341, 359-366, 372-378, 391-392, 397-399** and **433-434.**

We hope that these changes satisfy the concerns of Reviewer 3. Thank you again for your time reviewing this work. Please do contact me at [alexandria.innes@city.ac.uk](mailto:alexandria.innes@city.ac.uk)  if there is anything further we can provide.

We will look forward to hearing from you in due course.

Sincerely yours,

Alexandria Innes
